# An association-adjusted consensus deleterious scheme to classify homozygous Mis-sense mutations for personal genome interpretation

**DOI:** 10.1186/1756-0381-6-24

**Published:** 2013-12-23

**Authors:** Thanawadee Preeprem, Greg Gibson

**Affiliations:** 1 School of Biology, Georgia Institute of Technology, Atlanta, GA 30332, USA

**Keywords:** Homozygous variant, Non-synonymous single nucleotide polymorphism, Personal genome interpretation, Variant prioritization, Protein structure analysis

## Abstract

**Background:**

Personal genome analysis is now being considered for evaluation of disease risk in healthy individuals, utilizing both rare and common variants. Multiple scores have been developed to predict the deleteriousness of amino acid substitutions, using information on the allele frequencies, level of evolutionary conservation, and averaged structural evidence. However, agreement among these scores is limited and they likely over-estimate the fraction of the genome that is deleterious.

**Method:**

This study proposes an integrative approach to identify a subset of homozygous non-synonymous single nucleotide polymorphisms (nsSNPs). An 8-level classification scheme is constructed from the presence/absence of deleterious predictions combined with evidence of association with disease or complex traits. Detailed literature searches and structural validations are then performed for a subset of homozygous 826 mis-sense mutations in 575 proteins found in the genomes of 12 healthy adults.

**Results:**

Implementation of the Association-Adjusted Consensus Deleterious Scheme (AACDS) classifies 11% of all predicted highly deleterious homozygous variants as most likely to influence disease risk. The number of such variants per genome ranges from 0 to 8 with no significant difference between African and Caucasian Americans. Detailed analysis of mutations affecting the APOE, MTMR2, THSB1, CHIA, αMyHC, and AMY2A proteins shows how the protein structure is likely to be disrupted, even though the associated phenotypes have not been documented in the corresponding individuals.

**Conclusions:**

The classification system for homozygous nsSNPs provides an opportunity to systematically rank nsSNPs based on suggestive evidence from annotations and sequence-based predictions. The ranking scheme, in-depth literature searches, and structural validations of highly prioritized mis-sense mutations compliment traditional sequence-based approaches and should have particular utility for the development of individualized health profiles. An online tool reporting the AACDS score for any variant is provided at the authors’ website.

## Background

Personal genome interpretation is a process of determining the personal genome sequences and assessing the likely consequences of an individual’s genetic variation. Personalized genome data interpretation can be used, for example, to predict diseases and traits, identify mutations for family planning purposes, and guide medical treatments based on likely drug responses. Developments in next-generation sequencing technologies over the past five years have enabled personal genome interpretation to become feasible and affordable [1]. Despite these advances, understanding of the impact of specific genetic variants remains limited. Major efforts have been made to identify nsSNPs with strong effects because of their collective high prevalence and likelihood that many may be clinically actionable [2].

Sequence-based prediction algorithms are commonly used to categorize nsSNPs into damaging and non-damaging, and to predict the effects (small or large) of nsSNPs with respect to undesirable phenotypes. The algorithms score the amino acid changes from the level of sequence conservation observed in homologous sequences or from the degree of physicochemical changes. Structure-based predictions evaluate three-dimensional (3D) structural features, e.g., solvent accessibility, stability, number of residue contacts, which are altered in the mutant proteins. Approaches that include annotation of biological function also support functional assessments of amino acid substitutions. Ng and Henikoff (2006) proposed that the combination of all three types of data may provide the most accurate assessment of likely deleteriousness [3], which motivates the development of the schema proposed in this study.

A pioneer study in personal genome interpretation stated that the human reference genome carries 1104 nsSNPs *predicted* to have impact on protein functions [4]. A similar study indicated that there are 796–837 *predicted* deleterious nsSNPs per individual [5]. This number of *predicted* damaging nsSNPs is much greater than both the *theoretically estimated* 15–60 damaging nsSNPs per genome [2] and the *classified* disease-causing nsSNP number of 40–100 per genome [6]. These observations highlight the complexity of personal genome interpretation and the need for a variant classification schema that builds on algorithmic prediction by integrating sound knowledge of the biological and structural impact of genetic variants.

**Figure 1:**
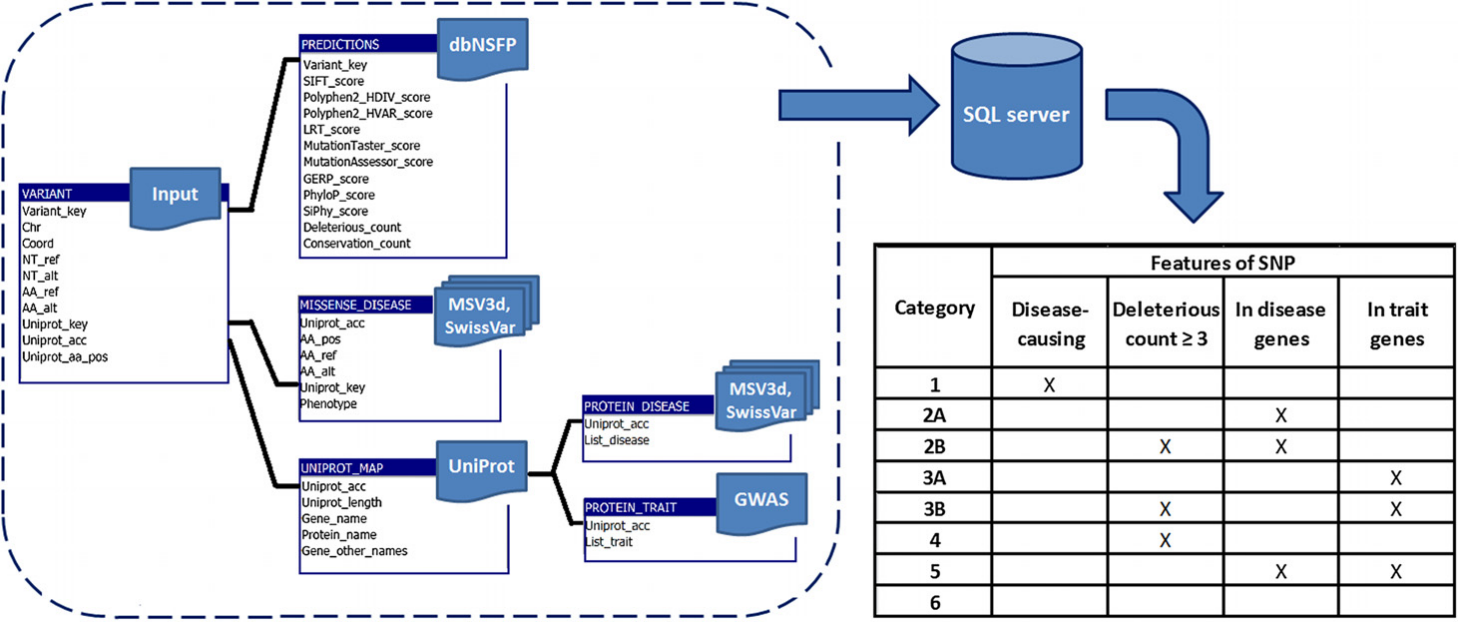
**Flow diagram for AACDS classification algorithm.** Upon receiving a list of homozygous rare mis-sense variants, the nsSNPs were mapped to the corresponding amino acid residue within a reference protein (identified with UniProt accession number). We use an SQL server to hold SNP-related information from several resources: deleterious predictions (from dbSNFP database [10]), known diseases associations of each variant and known disease/trait associations of each gene (from MSV3d [14], SwissVar [15], and GWAS databases [17]). The AACDS classification algorithm extracts relevant fields from the AACDS database to populate the report on the variant category (categories 1–6). The output (Additional file 1: Figure S1) is converted from an SQL data table to the user-defined formats (HTML, text), and is available for download, or individual queries can be supported on our server at http://www.cig.gatech.edu/Tools.

There are many databases that provide useful information about genetic variants. Because genetic polymorphisms found in healthy individuals tend to have small effects, further improvement of available resources is required to more accurately define the set of variants that are likely to be most important for an individual’s health. In this study, we constructed a classification schema (Figure 1, Additional file 1: Figure S1) to rank nsSNPs identified in healthy individuals by their functional significance. Each ranking category reflects the strength of evidence that a variant may adversely affect gene function from several standpoints, incorporating both database searches and sequence-based predictions. The newly developed variant classification scheme is designed to generate a best estimate of clinical significance for each variant of interest, with the intention of focusing attention on the most likely deleterious SNPs.

Given the very large number of candidate disease-promoting variants per genome, we here focus just on the homozygous variants reasoning that highly penetrant effects are most likely to be recessive. The methods developed could be applied to all heterozygous nsSNPs as well, but this would be a daunting task for manual inspection, which would only be warranted given extensive phenotype data and a desire of an individual to receive the information. Here we describe homozygous nsSNPs in the genomes of 12 healthy participants in a predictive health study, the Emory-Georgia Tech Center for Health Discovery and Well Being (CHDWB). Since the IRB consent does not allow communication of genetic data, given potential negative consequences of knowledge of variants that cannot be acted upon, the identities of the individuals are anonymous and no concerted attempt has been made here to link genotypes to phenotypes directly. Expanded and appropriately consented studies will be required to evaluate the actual utility of the proposed schema as a means of focusing attention on those variants that are most likely to influence personalized health behaviors.

## Methods

### Whole genome sequence dataset

Whole genome sequence (WGS) data was obtained for 12 healthy adult participants in the Center for Health Discovery and Well Being (CHDWB) study, including 4 African American women, 4 Caucasian women, and 4 Caucasian men. None of the individuals has any known complex or Mendelian diseases, but they cover a variety of profiles with respect to overall physical and mental health. Prediction of disease risk based on common variants and clinical profiles is described for the Caucasians in [7]. The participants have provided written consent to publication of their whole genome sequence data for research purposes only. They do discuss their clinical profile with a health professional following annual visits to the Center, but are not currently permitted to receive the genetic data generated during the study. In order to protect participant identities, their identifying numbers have been randomized for this study.

WGS was performed on Illumina HiSeq2000 automated sequencers at the University of Washington facility under contract to the Illumina Genome Network. Approximately 125 billion bases passing the Illumina analysis filter were obtained for each genome. Mean non-N reference coverage (after excluding gaps) is ~36X with 95.5% of the positions having an average coverage of at least 10X. The genome sequences were aligned against the human reference genome assembly (hg19 sequence) using CASAVA (Consensus Assessment of Sequence And VAriation, Illumina, Inc, San Diego, CA). On average, 87% of each individual’s quality filtered reads were aligned. High-confidence variants with a quality score above 20 were retained. Accuracy of the generated genome sequences was confirmed by comparison with previously determined genotypes from Illumina OmniQuad arrays, which showed >99% concordance for all individuals.

The coding variants were functionally annotated using Variant Annotation Tool (VAT) [8] which uses the GENCODE v7 gene annotation set [9]. We identified “homozygous rare variants” with allele frequency <10% in the Caucasian or African 1000 Genomes dataset, using the data provided by dbNSFP [10]. All homozygous rare nsSNPs relative to hg19 were identified for each person, and assigned to two categories: known nsSNPs that are present in dbSNP build 137 [11], and private nsSNPs that are absent from dbSNP but found exclusively in each individual. Information on the number of variants of each type is provided in Table 1. Minor allele frequencies (MAFs) for all nsSNPs were obtained from NHLBI GO Exome Sequencing Project (ESP6500) (June 2012 release) [12]. Genomic data for known homozygous nsSNPs (n = 826, including 29 private variants) were analyzed as a whole and per individual.

**Table 1:** Summary of genetic variations in genome sequences of 12 individuals

**Subject ID**	**Eth, sex**	**Total variants**			**Coding variants**								**Homozygous nsSNPs**			
		***(>q20)***			***(Based on gencode v7)***								***(Based on dbSNP build 137)***			
		**SNPs**	**Indels**	**SVs**	**SNPs**				**Indels**			**SVs**	**#Known nsSNPs**	**#Unique genes**	**#** ***de novo *** **nsSNPs**	**#Unique genes**
					**S**	**MS**	**NS**	**Splice**	**Indels**	**Indels**	**Indels**	**SVs**				
								**overlap**	**FS**	**NFS**	**overlap**	**overlap**				
1	Afr, F	4513763	733596	4251	14793	14039	72	98	381	342	137	37	88	77	2	2
2	Afr, F	4472988	754399	4545	14500	13712	66	106	393	335	147	55	71	63	3	3
3	Afr, F	4287739	722922	4447	13755	13166	84	79	374	301	120	43	77	71	3	2
4	Afr, F	4443799	746111	4368	14488	13874	73	104	366	338	142	40	58	56	1	1
5	Cau, F	3734820	645032	3977	11929	11745	62	90	343	307	123	43	57	40	2	2
6	Cau, F	3691337	633475	4114	11757	11457	56	90	317	280	106	49	52	45	None	None
7	Cau, F	3691270	632544	4033	11912	11488	65	71	279	304	116	37	50	44	4	4
8	Cau, F	3722234	641792	4197	11887	11434	64	76	303	299	125	41	55	42	None	None
9	Cau, M	3647944	590064	3828	11619	11255	54	76	311	281	95	38	60	50	2	2
10	Cau, M	3643046	597363	4011	11814	11480	61	85	289	287	109	31	82	65	2	2
11	Cau, M	3650690	602744	3916	11560	11285	60	80	342	280	112	32	72	65	5	5
12	Cau, M	3701558	639005	4739	11842	11708	60	81	334	290	118	37	75	64	5	5
													**#Total = 797**	**#Unique = 575**	**#Total = 29**	**#Unique = 25**

*Abbreviations*: *Eth* ethnicity, *SNPs* single nucleotide polymorphisms, *SV* structural variants, *S* synonymous, *MS* mis-sense, *NS* nonsense, *FS* frameshift, *NFS* non frameshift.

Amino acid indices for the alternate residues were mapped to the corresponding proteins using transcript IDs for the major isoform. All protein sequences and related information including protein functions and sequence features were obtained from the UniProt database [13].

### Sequence annotation using published algorithms

Evidence for association of each SNP or gene with diseases or traits was obtained from public repositories of amino acid polymorphisms (MSV3d, July 2012 release [14] and SwissVar, accessed December 2012) [15], from Online Mendelian Inheritance in Man (OMIM) [16], and from the NHGRI genome wide association studies (GWAS) [17] catalog. Initially, each nsSNP was assigned as disease-causing, probably disease causing, unclassified, or neutral. Functional predictions, and information on disease- and trait-associations to the gene were collected from dbNSFP [10]. In addition, we used UniProt sequence feature records [13] to annotate whether the mutated amino acid is localized to any structurally/functionally important sites (molecule processing sites, binding sites, modification sites, etc.).

To annotate deleterious nsSNPs, consensus predictions from several algorithms were compared. Pre-computed deleterious scores for each nsSNP were retrieved from dbNSFP [10]. To our knowledge, dbNSFP is the first database that provides pre-computed functional predictions from multiple algorithms, facilitating interpretation of the deleteriousness of variants in large datasets. The database provides the output of six different prediction algorithms that have complimentary methodologies. Three are sequence-based (SIFT [18], LRT [5], and MutationAssessor [19]), while two are both sequence and structure-based (PolyPhen2_HumDiv and PolyPhen2_HumVar [20]), and the sixth is the MutationTaster Bayesian classifier [21]). Each of these tools relies primarily on the basic assumption that residue functionality dictates sequence conservation, which can consequently be used to infer deleteriousness. Raw scores for the first five programs were re-scaled to [0, 1] in which a score closer to 1 represents a stronger (deleterious) effect of a nsSNP [10]. A MutationAssessor score of > 3.5 designates high functional impact [19], hence, “deleterious”. The six prediction programs were used to construct the classification scheme of nsSNPs presented in Table 2. Putative deleterious nsSNPs were identified as nsSNPs reported as “deleterious” by at least three out of six prediction programs.

**Table 2:** AACDS classification of homozygous nsSNPs in 12 genomes

		**Features of SNP**				**Subject id, ethnicity, sex**												**Total #nsSNPs**
		**Disease-causing**	**Deleterious count ≥ 3**	**In disease genes**	**In trait genes**	**1**	**2**	**3**	**4**	**5**	**6**	**7**	**8**	**9**	**10**	**11**	**12**	
						**Afr, F**	**Afr, F**	**Afr, F**	**Afr, F**	**Cau, F**	**Cau, F**	**Cau, F**	**Cau, F**	**Cau, M**	**Cau, M**	**Cau, M**	**Cau, M**	
**Category**	**1**	X				1	None	None	None	1	None	None	1	1	None	1	None	**5**
	**2A**			X		8	7	6	5	6	5	11	7	12	7	10	9	**93**
	**2B**		X	X		1	None	None	None	2	2	4	1	2	None	2	None	**14**
	**3A**				X	9	24	17	7	6	10	9	10	5	13	16	10	**136**
	**3B**		X		X	1	5	None	none	1	1	3	4	2	2	None	2	**21**
	**4**		X			5	5	8	6	None	5	3	7	4	6	5	4	**58**
	**5**			X	X	14	21	20	10	8	12	9	11	13	18	20	14	**170**
	**6**					68	40	49	42	46	32	33	33	40	56	45	55	**539**
**Total #unique nsSNPs**						**88**	**71**	**77**	**58**	**57**	**52**	**50**	**55**	**60**	**82**	**72**	**75**	**797**

The number of homozygous nsSNPs per individual and the number of SNPs in each AACDS category are specified**.**

In addition to the deleterious predictions, protein regions under evolutionary constraint were detected using three evolutionary conservation-based indicators: GERP++ [22], phyloP [23], and SiPhy [24]. We also used Grantham scores [25] to reflect the degree of physicochemical differences between pairs of amino acids. These four indicators were included in the analysis for comparison purposes but were not utilized for nsSNP categorization.

Other popular variant annotation tools might also be useful but were considered to be redundant with respect to our purposes. For example, ANNOVAR [26] has the ability to perform variant annotation (intronic, intergenic, untranslated region, exonic: non-synonymous, synonymous, etc.), but this information was already available from the VAT output. At the time of our analysis, ANNOVAR provided dbSNP build 135 mapping, not the dbSNP build 137 [11] used in our analysis pipeline. Note that gene definitions from ANNOVAR refer to the nucleotide reference sequence where a SNP is located; the format is not directly applicable for working with records from the two selected SNP databases (MSV3d [14] and SwissVar [15]), in which UniProt accessions were used to identify gene products. Similar to ANNOVAR, SnpEff [27] is another popular program that can assign structural annotations of variants. This function would be valuable when one wants to analyze different types of genetic variations within a genome. Because we only focused on analysis of mis-sense mutations, the annotation feature of SnpEff was deemed unnecessary.

### Supervised and automated structure-based predictions of variant function

High quality protein 3D structures are essential to identify functional impacts of nsSNPs. Due to the limited availability of experimentally-determined human protein structures [28], an assortment of 3D structure sources was used to manually evaluate the effects of single point mutations found in specific proteins (Additional file 2: Table S1). Crystal structures were retrieved from the RCSB Protein Data Bank (PDB) [29]. Homology models were retrieved from Protein Model Portal (PMP) [30] repository, or were built manually by joining multiple structures into a single model of a protein. Steric conflicts found within homology models were resolved by energy minimization with explicit solvent using YASARA force field [31]. Structural validation of homology models was evaluated by using two independent scores: QMEAN6 [32] and ModFOLD4 [33]. All 3D structures were visualized and rendered using Chimera [34].

The analysis began with a visualization of wild type proteins in the context of bound ligands. Additional variants that are known to be associated with diseases, or affect protein functionality and/or stability were also identified for each protein structure. Next, we used SDM [35] to compare the protein stability changes upon amino acid mutations with the default modeling of a mutant structure using Andante [36]. A mutation is classified as affecting protein function (stabilizing or destabilizing) using the stability cutoff of ±2 kcal mol^-1^[35]. For evaluating the impact of amino acid changes on protein stability in a high throughput fashion, we obtained the tertiary classification of protein stability changes (increase, decrease, neutral) caused by a SNP from I-Mutant 2.0 [37], available from MSV3d [14]. The predictions are based on the protein (or homolog) structure or solely on the protein sequence when the structure was unavailable.

In order to expand the structure-based predictions to a larger dataset without the requirements of manual inspection or in-depth literature searches, we applied a combination of database searches and computational predictions to a larger set of proteins. In addition to the 6 protein structures described in the main text, we identified an additional 25 protein coordinates from PDB [29] (Additional file 2: Table S2). We assessed these 25 wild type proteins in 4 areas of structural analysis: protein stability, ligand binding capability, protein dynamics, and protein-protein interactions. For protein stability, we used the aforementioned approach along with predictions of amino acids with specialized roles regarding protein stability, namely long-range stabilization center (SC) residues and stabilizing residues (SRs). These residues were inferred from the SCide [38,39] and SRide webservers [40], respectively. Ligand binding residues for each protein were retrieved from PDBe (http://www.ebi.ac.uk/pdbe/) or were predicted using 3DLigandSite [41]. Amino acid residues that are located in or near predicted binding pockets are likely to alter the binding capability for ligand(s). As disease-causing mutations that do not occur in binding sites or buried sites are predominantly found on protein interfaces [42], we used the PatchFinder program [43,44] to computationally predict the most significant cluster of conserved residues on a protein’s surface that may indicate possible functional sites of the protein; i.e., sites of protein-protein interactions. Changes in protein dynamics were evaluated by the crystallographic B-factor of Cα atoms. In addition, we also used PredyFlexy [45] and FlexPred [46,47] to predict the dynamic class of an amino acid residue (rigid, intermediate, flexible), and to estimate whether each residue is likely to induce conformational switches within the protein.

### Assessment of functional enrichment

The g:Profiler web server [48] was used to detect enrichment of gene functions for genes whose nsSNP are homozygous. Functional profiling and statistical enrichment analysis were performed with two distinct methods. First, we compared the annotations of multiple gene lists, where each list represents the genes with known homozygous variants found in an individual, using G:Cocoa (n = 40–77 genes per genome). Then, we analyzed a gene list for each individual using G:GOSt. The second analysis was performed in two-steps: with and without genes harboring private variants. Enriched functions, such as common gene ontology, biological pathways, shared transcription factor or miRNA binding sites, were reported using the default g:SCS method for significance threshold determination. It is worth mentioning that significant enrichment of protein-protein interactions, derived from the BioGRID database [49], does not imply that all genes with significant enrichment p-value are interacting with each other, but simply indicates which query genes are present in the entire BioGRID dataset. The actual number of interactions and associated genes can be visualized from the network output. The enriched annotations and their gene members were confirmed by literature searches. Furthermore, the SNPshot text-mining tool for PubMed abstracts was used to explore if any of the private homozygous nsSNP-containing genes have clinical or experimental evidence for gene-drug or gene-disease associations [50].

## Results and discussion

### Sequence-based variant description in 12 genomes

A total of 797 known homozygous non-synonymous substitutions was observed in 575 different genes (Table 1). The genomes of the four African individuals harbor on average 73 homozygous nsSNPs (range 58–88), while the eight Caucasian genomes have an average of 63 homozygous nsSNPs (range 50–82). The slight excess in African Americans is not statistically significant (*p* = 0.18, 2-tailed t-test). 456 of the genes (79%) have a single homozygous variant in the 12 genomes, but two genes (*HLA-DRB5* and *ANKRD20A4*) have more than 10, detected in at least 6 individuals.

The vast majority of all of the variants have been observed previously in the 1000Genomes project, with just 29 private homozygous nsSNPs observed in 25 different genes, with a range of 0 to 5 per genome. Private variants were found in 10 out of 12 genomes of CHDWB dataset and are listed in Additional file 2: Table S3. Most are predicted to be neutral, though they affect nucleotides with a range of conservation levels. Almost one-third of the 25 genes have no defined functions, and only a minor proportion of the genes have been previously associated with a disease or trait.

Among the 575 genes whose nsSNPs are homozygous and present in dbSNP build 137, the fractions with known functions, putative functions, and unknown functions are ~47, 20, and 33%, respectively (Table 3). Almost 10% of the homozygous nsSNPs are found in four highly represented protein groups: transcriptional regulators, keratin-associated proteins, odorant receptors, and zinc finger-containing proteins.

**Table 3:** Functional annotation of all homozygous nsSNP

**Gene groups**	**Genes**		**nsSNPs**		**Most common proteins**
	**#**	**%**	**#**	**%**	**(#proteins, #nsSNPs)**
Genes with known functions	268	47%	340	43%	Transcriptional regulator proteins (15 proteins, 17 nsSNPs), Keratin-associated proteins (6 proteins, 9 nsSNPs)
Genes with putative functions	118	20%	167	21%	Potential odorant receptors (21 proteins, 27 nsSNPs), Zinc finger-containing proteins, potentially for transcriptional regulations (16 proteins, 22 nsSNPs)
Genes with unknown functions	189	33%	290	36%	-
Total	575	100%	797	100%	

**Figure 2:**
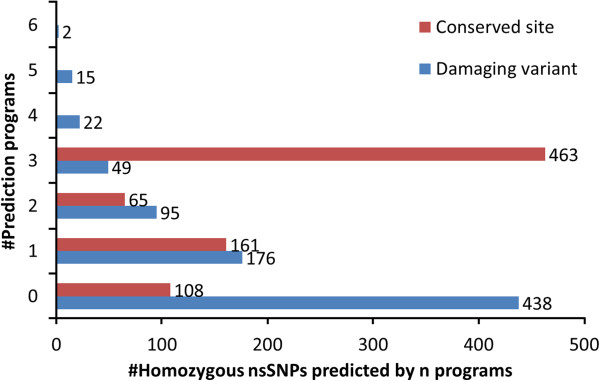
**Number of deleterious and conserved site predictions.** Data labels indicate the numbers of homozygous nsSNPs predicted to be damaging or conserved by *n* programs (*n* = 0-6 for deleterious predictions, and *n* = 0-3 for conservation predictions).

Six programs were used to predict deleterious variants, and three to indicate the level of sequence conservation at the polymorphic site. The results are summarized in Figure 2, which shows the cumulative number of predicted deleterious (blue) or highly conserved (red) scores for each of the 797 variants. Although 58% of all homozygous mis-sense variants in the 12 genomes alter conserved sites in all three assessments, almost 40% of these (183/463) are predicted to be functionally neutral by all six programs. 45% of the homozygous rare variants are predicted deleterious by at least one program, and 11% (88 variants) by three or more programs.

**Figure 3:**
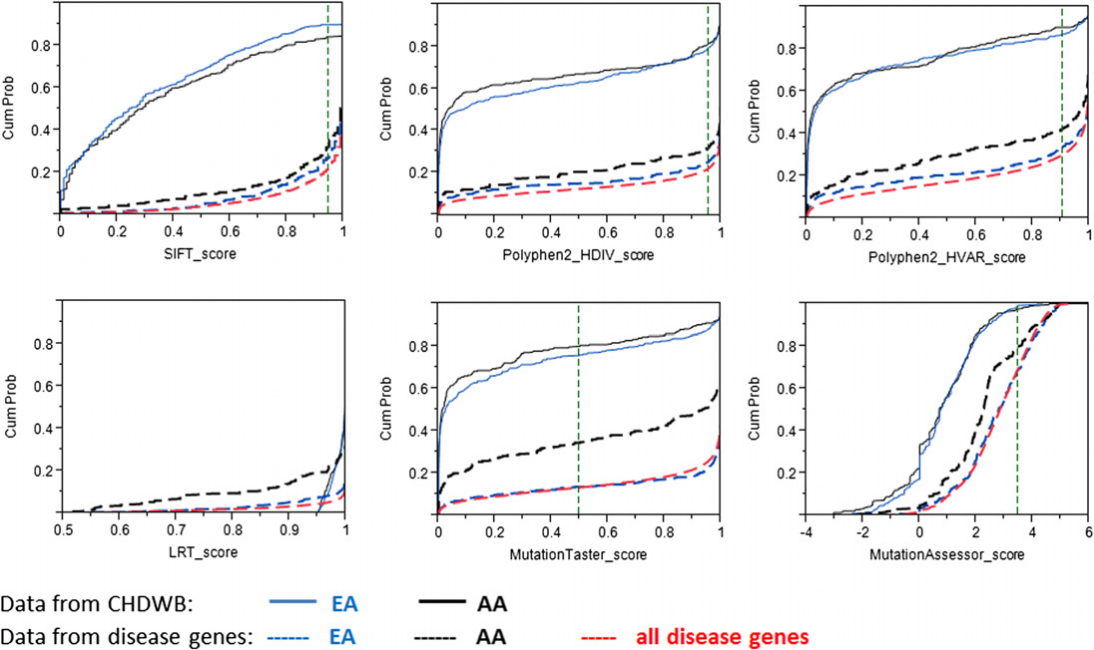
**Cumulative distribution plots for the six deleterious prediction scores.** The X-axis represents the prediction scores, ordered by the deleteriousness. The lowest and the highest scores for each prediction algorithm indicate the neutral and damaging nsSNP, respectively. For each prediction program, the score threshold for defining damaging SNPs is indicated by a vertical dashed green line (threshold for LRT is at 0.999). Five sets of SNP data are shown in each plot. Black solid lines: data from homozygous nsSNPs of four African individuals; blue solid lines: data from homozygous nsSNPs of eight Caucasian individuals; red dashed lines: data from all disease-causing nsSNPs (n = 24703 nsSNPs in 1789 proteins); black dashed lines: data from disease-causing nsSNPs found in homozygous nsSNP-containing genes of the four African individuals (n = 348 nsSNPs in 23 genes); blue dashed lines: data from disease-causing nsSNPs found in homozygous nsSNP-containing genes of the eight Caucasian individuals (n = 547 nsSNPs in 44 genes). All disease-causing nsSNPs were retrieved from MSV3D [14] and SwissVar [15].

Differences among the deleteriousness prediction algorithms are underscored by the cumulative score distribution plots in Figure 3. The solid lines in the top half of each plot are for the 294 homozygous rare variants in the African Americans (black) and 503 homozygous rare variants in the Caucasians (blue). The two curves are not significantly different, and predict that as many as 28% of variants are deleterious (MutationTaster) or as few as 2% (MutationAssessor), with the two most commonly used algorithms, SIFT and PolyPhen2, giving intermediate estimates of 80% neutral. The lower curves show cumulative distributions for a set of 24703 disease-promoting non-synonymous variants in 1789 proteins compiled from the MSV3d [14] and SwissVar [15] databases (red dashed curve), as well as from subsets of these disease variants found only in the 23 genes (348 SNPs) harboring homozygous nsSNPs in the four African Americans, or 44 genes (547 SNPs) in the eight Caucasians in our sample (black and blue-dashed curves respectively). All six programs show an elevated tendency to predict known disease-associated variants in the genes harboring homozygous variants in the African Americans as neutral. This is particularly obvious for the MutationTaster score and least pronounced for SIFT. A similar observation of differences among Asians, Caucasians and Africans in the fraction of damaging SNPs predicted deleterious was made by [51].

The notable differences in deleterious predictions given a set of disease-promoting variants found in African American and Caucasian samples (Figure 3, lower curves) suggest these prediction algorithms may have population-specific effects. To further investigate that this observation is not an artifact of the small samples, we plotted similar curves for a set of 1789 genes harboring disease SNPs (total 24703 SNPs). The genes were classified into four groups depending upon population bias of their SNPs, using %MAF difference cutoff of ± 5% between European American (EA) and African American (AA) populations. The four gene groups are: EA bias, AA bias, EA&AA bias, and no bias. Unlike Figure 3 where homozygous variants in the African Americans were consistently predicted to be more neutral, the larger-sample plots (Additional file 3: Figure S2) illustrate a small difference in the cumulative scores of population specific-disease SNPs, with the exception of a noticeable prediction bias of MutationTaster. The results highlight the need for development of mutation assessment pipelines that go beyond these algorithms, particularly when evaluating homozygous nsSNPs of non-Caucasian genomes.

### Association-adjusted consensus deleterious scheme (AACDS) for variant classification

Consequently, we developed a ranking system that classifies homozygous nsSNPs into eight categories according to the overlap of (i) consensus deleterious prediction, (ii) documentation that the SNP causes a disease, and evidence that the SNP is in a gene that has been associated with (iii) a disease or (iv) a quantitative trait (Figure 1). Category 1 contains documented disease-causing nsSNPs. Categories 2A and 3A represent nsSNPs in genes that have known associations with diseases (2A) or traits (3A), and these are sub-divided into categories 2B and 3B if they are also predicted to be deleterious by three or more programs. Category 4 comprises nsSNPs that are predicted to be damaging but lie in genes that have no clinical associations. Conversely, Category 5 nsSNPs are located in genes that have disease or trait associations, but variants are predicted to be neutral. Category 6 represents neutral nsSNPs, whose genes have no clinical relations. Table 2 lists the number of variants in each category from each individual’s genome.

The list of disease-promoting nsSNPs was retrieved from MSV3d [14] and SwissVar [15] and is based on manual curation of evidence that the variant is causal, or probably causal, in disease. Most are relatively rare (MAF < 5%) presumed highly penetrant mutations, but an unknown fraction may be false positives. Among the 575 genes in our dataset, 93 harbor disease causal variants (n = 787 nsSNPs). The 797 homozygous nsSNPs in our dataset include 4 category 1 “disease-causing” mutations (1 SNP is present in two individuals) and another 6 listed as probably pathogenic (Table 4). Another 143 are classified as having unknown effects, leaving 644 polymorphisms presumed not to cause disease in a highly penetrant manner. The 10 probably or known pathogenic mis-sense variants were predicted to be deleterious by 0 to 5 programs (Table 4).

**Table 4:** List of all four known disease-causal variants and six probable pathogenic variants

**SNP type**	**Gene**	**Position, base change (AA change)**	**rsID (%MAF EA/AA/All)**	**Disease [prediction counts]**	**Grantham score**	**Protein stability change**	**Site annotations**
Disease-causal	*ATP6V0A4*	7:138417791	rs3807153* (4.8/18.5/9.4)	Distal renal tubular acidosis (dRTA) with preserved hearing	81	Neutral	TRANSMEM
		*A*-->*G (M580T)*		[Del count: 2; Con count: 3]			
	*MTMR2*	11:95569448	rs558018 (0.02/3.9/1.3)	Charcot-Marie-Tooth disease type 4B1 (CMT4B1)	46	Decrease	DOMAIN
		*T*-->*C (N545S)*		[Del count: 2; Con count: 3]			
	*APOE*	19:45412079	rs7412 (5.6/8.7/6.6)	Lipoprotein glomerulopathy (LPG)	180	Decrease	REPEAT
		*C*-->*T (R176C)*		[Del count: 5; Con count: 3]			
	*BMP15*	X:50658966	rs104894767 (1.4/0.3/1.0)	Premature ovarian failure type 4 (POF4)	58	Neutral	PROPEP
		*G*-->*A (A180T)*		[Del count: 0; Con count: 1]			
Probable pathogenic	*FRZB*	2:183699584	rs7775 (8.8/28.4/15.4)	Osteoarthritis type 1 (OS1)	125	Neutral	-
		*G*-->*C (R324G)*		[Del count: 1; Con count: 3]			
	*HABP2*	10:115348046	rs7080536 (3.9/0.7/2.8)	[Del count: 5; Con count: 3]	98	Decrease	DOMAIN
		*G*-->*A (G534E)*					
	*HNF1A*	12:121416650	rs1169288 (33.5/12.1/26.2)	Insulin-dependent diabetes mellitus type 20 (IDDM20)	5	Neutral	REGION (Dimerization)
		*A*-->*C (I27L)*		[Del count: 1; Con count: 3]			
	*XYLT1*	16:17564311	rs61758388 (-/-/1.7)	[Del count: 0; Con count: 3]	99	Neutral	TOPO_DOM
		*C*-->*A (A115S)*					
	*CYP2A6*	19:41354533	rs1801272 (2.5/0.5/1.8)	[Del count: 1; Con count: 3]	99	Decrease	-
		*A*-->*T (L160H)*					
	*ADA*	20:43255220	rs11555566 (6.3/6.8/6.5)	Severe combined immunodeficiency autosomal recessive T-cell-negative/B-cell- negative/NK-cell-negative due to adenosine deaminase deficiency (ADASCID)	26	Decrease	-
		*T*-->*C (K80R)*					
				[Del count: 1; Con count: 3]			

*The first SNP (rs3807153) was observed in two individuals.

The minor allele frequency (MAF) in percent listed in the order of European American (EA), African American (AA), and all populations (All), delimited by “/”. Prediction counts indicate the number of deleterious predictions (Del count) and conservation predictions (Con count) by six and three programs, respectively. Grantham score determines the similarity in amino acid changes: small (score < 60), intermediate (score 60–99), and large (score ≥ 100). Tertiary classification (increase, decrease, neutral) for protein stability change caused by a SNP was obtained from I-Mutant 2.0 [37], available from MSV3d [14]. Site annotations list any structurally/functionally important sites (molecule processing sites, binding sites, modification sites, etc.) where the altered amino acid residue resides. The information was retrieved from UniProt sequence feature records [13].

Among the 93 disease-associated genes with homozygous nsSNPs in our CHDWB genomes, 18 have homozygous variants present in more than one individual, and these account for 40 nsSNPs, observed at 33 different sites. Only one of these sites, *G56R* in the MYH6 myosin heavy chain, is predicted to be deleterious. Since it is also associated with resting heart rate, it is classified in both categories 2B and 3B. Data for the variant categorization in these 18 disease-associated genes is summarized in Additional file 2: Table S4. Similarly, we also observed 101 genes with trait associations, including 126 SNPs. Since there are a total of 14 and 21 variants in categories 2B and 3B respectively, most of these cases are restricted to a single individual in the sample of 12. Details for these predicted deleterious variants are listed in Additional file 2: Tables S5, S6, respectively. On average, each individual carries 3.33 category 1, 2B or 3B homozygous variants (range 0 to 7), and although the two individuals with no variants of this type are both African Americans, there is no significant difference in prevalence relative to the Caucasians (*p* = 0.21, 2-tailed *t*-test). All of the remaining predicted deleterious variant that do not have disease or trait associations (namely, category 4) are listed in Additional file 2: Table S7.

The set of 35 disease- or trait-associated SNPs that are also predicted to be deleterious is seven times larger than the set of 5 category 1 “known to be deleterious” mutations. They represent 15% of the 93 disease-associated and 136 trait-associated category 2A and 3A SNPs. Additional file 4: Figure S3 compares the allele frequency distributions of the 2B/3B SNPs relative to all 2A/3A SNPs and shows a tendency to reduced allele frequency, also consistent with them having deleterious effects on fitness. Another 170 homozygous nsSNPs lie in genes that have been associated with diseases or traits but are not predicted to be deleterious (category 5). Their frequency distribution is approximately equivalent to those of the category 2A and 3A SNPs. The vast majority (539) of the 797 SNPs we have considered are in category 6 and represent the subsets that are least likely to be damaging.

A limitation of our analysis is the uncertainty in the accuracy of phenotypic annotations of SNPs, as well as the variable confidence level in annotations of causal SNPs. We obtained the list of disease-promoting nsSNPs from MSV3d [14] and SwissVar [15]. Most of the variants were classified as either causal variants (to a specific disease), or as polymorphisms. In MSV3d, many variants have ambiguous annotations, e.g., probable-pathogenic, or unknown. In SwissVar, some proteins are noted to have associations with diseases, but the list of variants is not provided.

### Supervised structure-based variant evaluation

Personal genome studies indicate that each healthy individual carries a large number of rare homozygous genetic variants [52-55]. While these variants can be found in any structural regions along the genome and can have diverse effects on biological function, Cooper (2010) estimated that as many as 60% of known disease-causing mutations are nsSNPs [2]. This viewpoint simply reflects the more obvious impact of nsSNPs on coding regions than regulatory regions: the substitutions tend to alter the amino acid sequences of the proteins. Amino acid changes are thought to have profound effects on the protein, impacting their structure or function. Furthermore, it is believed that there exist some structure-function relationships for each individual amino acid residue within a protein chain, and the 3D structure is an ideal resource for investigating this information [56]. Therefore, many algorithms have been developed to assess the effects of amino acid changes within the context of protein 3D structures. Numerous structural features have been used to explain/quantify the changes [57]. A few successful implementations have demonstrated that protein 3D structures add to prediction accuracy [58,59]. In addition, *in silico* analysis of 3D structures can facilitate variant prioritization because it provides systematic screening of nsSNP effects in the context of the protein structure and suggests which mutations may critically alter the function of the protein.

Implementation of the AACDS classification scheme reduces the number of potentially deleterious variants to a number per genome that can feasibly be evaluated manually on a case-by-case basis. For this purpose, we have devised a further pipeline that involves sequence annotations, extracting either X-ray crystal/NMR structures or homology models from structure databases, and computing a series of predictions that capture protein features. In this way, each of the up to 5 variants in categories 1, 2B or 3B can be assessed in the context of the actual protein. While this approach requires that an individual with experience in protein structures be engaged in the personal genome evaluation, the potential gain in accuracy is likely to be meaningful.

**Figure 4:**
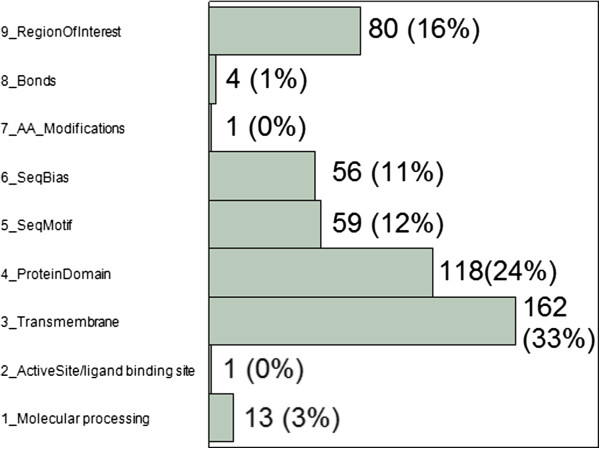
**Distribution of homozygous nsSNPs by sequence type.** Data labels indicate the number (and percentage) of SNPs altering protein residues with each specified sequence feature. All sequence features were obtained from the UniProt database [13].

Our preliminary analysis utilized sequence features for all amino acid residues in each protein, obtained from UniProt features records [13]. The entries had been curated and are predicted (and compatible with the protein function), experimentally proven, or determined by resolution of the protein structure. The analysis was restricted to 62% of the nsSNPs, since the remaining fractions do not currently have feature information. Figure 4 illustrates that although the annotated 494 nsSNPs are found in various sequence regions, they are predominately present in transmembrane and protein domains. These proportions are approximately equivalent to the proportions of each of the 9 types of annotated protein region for all residues in the included proteins (Additional file 5: Figure S4). Locations of homozygous variants relative to the length of each sequence feature indicate the variants are located throughout the entire sequence length (Additional file 6: Figure S5).

**Figure 5:**
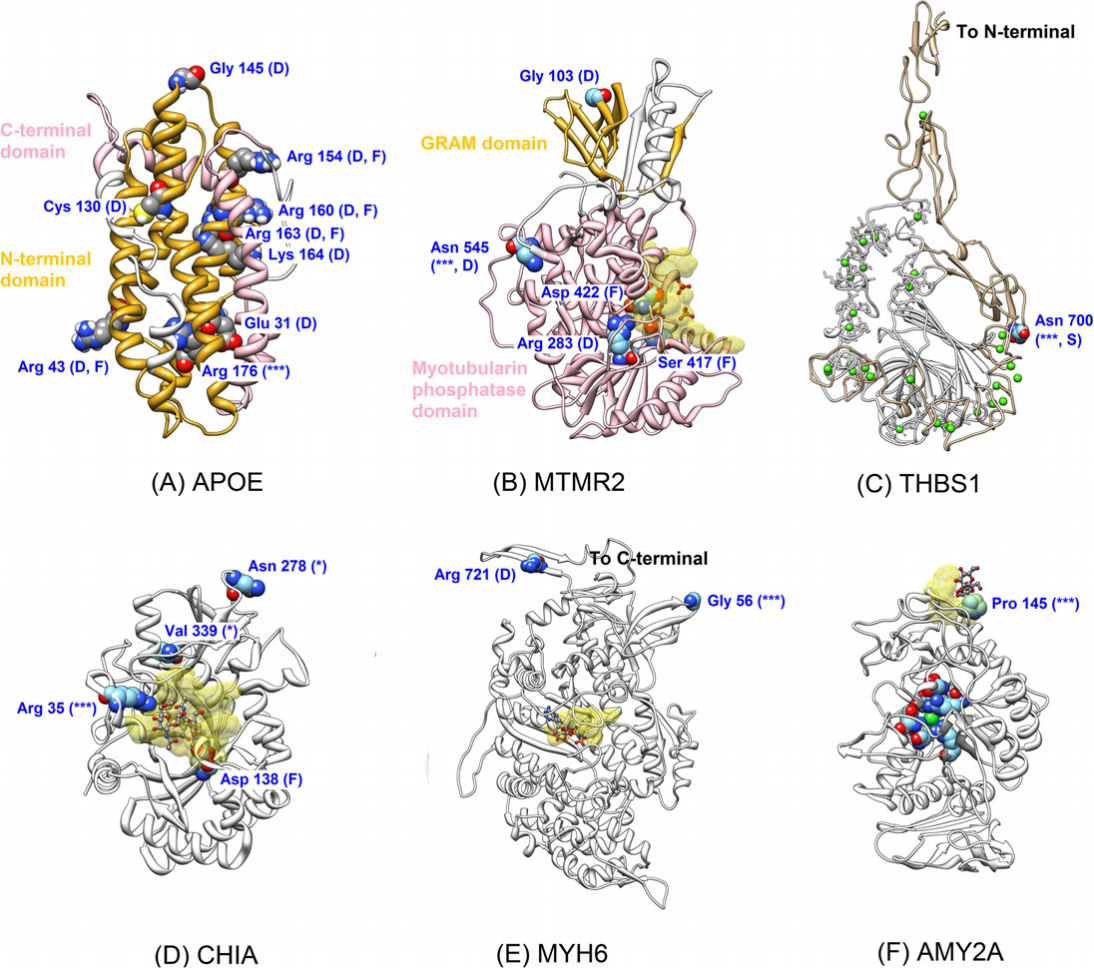
**Location of variants in six protein structures. A-B** describe two causative variants, **C-D** demonstrate two predicted deleterious variants, **E-F** illustrate two predicted deleterious variants whose clinical associations are inconclusive. For all figures, the representations are as follows: ribbon for proteins, ball and stick for ligands, mesh for ligand binding sites, and sphere for amino acid variants. Amino acid variants caused by homozygous or heterozygous nsSNPs are indicated as (***) or (*), respectively. Additional variants that are known to be associated with diseases (D), or affect protein functionality (F), and/or stability (S) are also identified. **A**: Apolipoprotein E (PDB:2L7B). **B**: Myotubularin-related protein 2 (PDB:1LW3). **C**: Thrombospondin-1 (PDB:1UX6 and homology model). Residues 1–548 are missing from the structure, residues 549–829 (brown ribbons) are modeled from human THBS2 (PDB:1YO8), residues 848–1169 (white ribbons) are from a crystal structure of THBS1 (PDB:1UX6). Coordination complexes of amino acid side chains and Ca^2+^ ions (green spheres) as seen in the crystal structures are indicated with purple lines. The Ca^2+^ ions with unidentified coordination complexes are derived from the superposition of Ca^2+^ ions in 1YO8 onto the homology model. **D**: Acidic mammalian chitinase (PDB:3FY1). Three homozygous nsSNPs were also identified from the same genome, but only two are present in the crystal structure. **E**: Myosin heavy chain 6 (αMyHC) modeled from Myosin heavy chain 7 (βMyHC) (PDB:4DB1). The C-terminal (residues 778–1939) is missing from the template crystal structure. **F**: Pancreatic alpha-amylase (AMY2A) (PDB:3OLE). The *Pro145* is located at the end of the extended β-loop and is part of a binding site for α-D-glucose. For clarity, only one α-D-glucose binding site is shown. Other variants known to affect the enzymatic activities are located around the chloride ion (green sphere) in the central vicinity of the protein.

Subsequent detailed analyses involved manual inspection and evaluation of individual proteins. The remainder of this section discusses detailed structural evaluations of two known causative variants, two predicted deleterious variants in proteins that have been associated with a disease or trait, and two predicted deleterious variants for which the clinical associations are inconclusive. The first disease causing nsSNP is the well-known Arginine to Cysteine substitution at residue 176 (residue 158 if omitting the signal peptide) that defines the *APOE2* allele of Apolipoprotein 2 (SNP category 1/2B/3B). This allele has a major influence on lipid transport and is a protective factor against late-onset Alzheimer’s disease and coronary artery disease [60-62]. Homozygosity for *R176C* is also associated with Type III hyperlipoproteinemia (HLPP3) in approximately 2% of cases (though 94% of HLPP3 cases have the genotype) [63]. Onset of the disorder is usually only after menopause in women and rarely manifests before the third decade in men. Several other rare variants in the gene have been annotated to disease, most of which affect intra- and inter-helical salt bridges (Figure 5A). With the neutral cysteine at position 176 in APOE2 protein, this pattern of salt bridge is eliminated. Although *Arg176* does not interact with the LDL receptor, the *R176C* substitution has been shown to indirectly reduce the receptor-binding activity of APOE [64]. Stability prediction indicates this mutation has neutral effect to the protein stability (ΔΔG = −0.46 kcal mol^-1^).

The second example of causative mutation is in Myotubularin-related protein 2 (MTMR2), a putative tyrosine kinase that is associated with Charcot-Marie-Tooth disease type 4B (CMT4B) [65]. One African individual has an Asparagine to a Serine substitution at the position 545 of the protein that has previously been reported as a rare variant in patients with CMT disease [66]. This SNP is classified as category 1. The minor allele frequency of this nsSNP is reported as 3.88% and 0.02% in African Americans, and European Americans, respectively, so the penetrance is much reduced in African Americans since disease prevalence of all forms of CMT is just one in 2500 [67]. The variant is situated in a conserved site, but only two algorithms predict it to be deleterious. The crystal structure places *Asn545* in a protein domain, but it is not in close proximity with two other causative variants or part of a binding site. This mutation is also predicted to be neutral (ΔΔG = 0.36 kcal mol^-1^) (Figure 5B).

One predicted damaging nsSNP was found in Thrombospondin 1 (THBS1), a glycoprotein that stabilizes fibrinogen platelet cross-bridges [68]. The homology model indicates the substitution of a Serine for Asparagine at residue 700 of THBS1 occurs at a critical position in one of the calcium-binding domains (green spheres project coordination of CA^2+^ ions) (Figure 5C). This *Asn700Ser* substitution in THBS1 (SNP category 4) has a prevalence of 8-10% in Europeans [69] and is associated with the occurrence of premature (age < 45) coronary heart disease in both homozygous and heterozygous individuals [68]. However, a study of *Asn702Ser* in THBS2 (homologous to 700 in THBS1) demonstrated that this variant does not alter calcium-binding capability. Instead, it induces a local conformational change leading to destabilization of surrounding structures [69], consistent with the computational prediction that the variant has a destabilizing ΔΔG of 0.58 kcal mol^-1^.

The second example of a predicted deleterious nsSNP is a mis-sense substitution in Acidic mammalian chitinase (CHIA), an enzyme that stimulates chemokine production by pulmonary epithelial cells. *Arg35Trp* (SNP category 4) is located in a buried site, where it causes changes in residue side chain volume, charge, polarity, and hydrophobicity (Figure 5D). The substitution was predicted to disrupt the hydrophobicity of the protein and increase solvent accessibility of the residue [14]. The individual who is homozygous for this variant also carries three heterozygous nsSNPs (*N278D*, *I339V*, and *V432G*). The first two of these replacements are parts of disulfide bonds, while the third substitution resides in the chitin-binding domain. Interestingly, the *Arg35Trp* mutation is predicted to stabilize the protein (ΔΔG = −1.19 kcal mol^-1^), a finding that may appear counter-intuitive. However, it is suggested that protein flexibility is crucial for enzyme catalysis [35]. The increase in protein stability and the dramatic change in physicochemical properties caused by this homozygous nsSNP, along with the disulfide bond reduction from heterozygous variants, strongly suggest the possibility for protein malfunction in this individual. As far as we are aware, he does not have asthma or an aberrant T-helper mediated inflammatory response, but deeper clinical investigation may be warranted.

The last two examples highlight cases where the variant is predicted deleterious but its clinical associations are inconclusive. The first example is a Glycine to Arginine substitution at residue 56 of Myosin heavy chain alpha (MYH6) (SNP category 2B/3B). As mentioned earlier, this is the only predicted deleterious variant among a set of 18 disease-associated genes with variants present in more than one individual (Additional file 2: Table S4). SNP databases indicate six well known causative variants in this gene that lead to familial hypertrophic cardiomyopathy and atrial septal defect. Although *G56R* is not one of them, this variant had been previously identified in affected individuals but it does not segregate perfectly with the disease in families of probands [70,71]. With regard to the homology model (Figure 5E), many of the known variants associating with heart disease are located in the coiled-coil regions of this protein (missing from the crystal structure) and are not part of the ATPase catalytic site or actin binding site. Nonetheless, the *G56R* found in one of the CHDWB participants is particularly interesting, since the mutation occurs in a myosin head-like domain, a key component for muscle contraction, and with a large degree of amino acid change (Grantham score = 125). Stability prediction also suggests this variant destabilizes the protein (ΔΔG = 1.10 kcal mol^-1^).

The second example of a variant with uncertain functional effect is taken from a set of 143 SNPs (18% of CHDWB dataset) that do not currently have phenotypic annotations. Among these, 117 SNPs have neither disease nor trait association at the gene level. Form 143 SNPs, we identified 15 variants predicted to be damaging, of which 8 are located in genes with no clinical associations. Our example is a Proline to Serine substitution at residue 145 of Pancreatic alpha-amylase (AMY2A) (SNP category 4). In addition to one calcium ion and one chloride ion per subunit, the protein is able to bind to several ligands throughout the structure. Mutagenesis studies identified several amino acid residues that are essential for the protein’s catalytic activity and affinity to calcium and chloride ions, but the impact of *Pro145Ser* has not been established [72,73]. Despite the limited information of the variant, crystal structure indicates *Pro145* is part of one, among many, binding sites for alpha-D-glucose (Figure 5F). In general, amino acids with similar physicochemical properties may substitute each other while maintaining the protein’s functionality. One study demonstrates some uncommonly predominant inter-species amino acid variations, such as serine-proline pairs or glutamic acid-alanine pairs [74]. The notable feature corresponds with the proline to serine substitution caused by this SNP. It is well known that the proline residue is sterically restricted and that it tends to disrupt regular secondary structural elements. Most proline residues are found in very tight turns or on protein surface [75]. The unusual occurrence of *Pro145*, especially in the extended β-loop indicates that this residue is essential for proper protein folding. Further investigation revealed that this residue is in the *cis* isomer, a very rare phenomenon since proline residues are exclusively synthesized as the *trans* isomer. In fact, AMY2A contains two *cis*-proline residues (*Pro69* and *Pro145*); both are located in the loop regions. It had been suggested that the two residues help accommodate a sharp turn of the β-loop [76]. Substitution of proline to serine is predicted to be highly stabilizing for residue 145 (ΔΔG = −2.77 kcal mol^-1^), but highly destabilizing for residue 69 (ΔΔG = 5.23 kcal mol^-1^). In any case, strong stability changes are suggested to cause protein malfunction and may lead to disease(s) [35].

### Automated structure-based variant evaluation

To facilitate high throughput evaluation of protein structures, we devised a structural analysis pipeline that assesses the functionality of protein residues using data directly obtained from the atomic coordinates or from computational predictions. Using this approach, a list of potentially deleterious variants from a structural perspective can be generated rapidly, providing a way to integrate structural analysis into the variant categorization scheme.

The four areas of automated structure-based variant analysis include stability, flexibility, and potential to disrupt protein-protein or protein-small molecule interactions. Many mutations disrupt these structural features and as a result, lead to altered protein functions or diseases. Our assumption is that the analysis may be able to identify some variants with strong effects. The results are summarized in Additional file 2: Table S8, which indicates that predicted deleterious variants show a wide variety of residue features. In general, SNPs that do not cause stability change (∆∆G < ± 0.5 kcal mol^-1^) tend to be non-deleterious, but not *vice versa*. Four amino acid residues have B-factors of Cα atoms larger than 60 Å^2^, a characteristic which may indicate that the atom is disordered due to dynamic motion, or may be an artifact of crystal imperfection. However, three out of these four amino acids are predicted to be at rigid sites and do not induce conformational switches. Another three residues are predicted to lie within a cluster of conserved residues on a protein surface, but none is categorized as damaging variants. Lastly, we found only one amino acid mutation that is located within the binding site, but the variant is in categories 2A and so not predicted to be damaging.

Imposing the constraint that the 3D structures must be of high quality, our initial analysis was restricted to only 24 experimentally-determined structures and 1 high quality theoretical model. Their sequence coverage ranges from 23–100% (average 69%) (Additional file 2: Table S2). However, the implementation can be further applied to any available structures. For example, using the automated Phyre2 homology modeling server with single/multiple template methodology [77], we were able to model an additional 77 full length proteins with high confidence. Each full length protein model has a percentage of residues modeled at >90% confidence in the range of 59-99% (average 86%). For larger proteins (>1300 amino acids), we truncated them into smaller domain(s) and our modeling attempt returned 10 models with confidence between 96-100%.

### Enrichment for mutations disrupting protein interactions

As a parallel approach to evaluating the deleterious potential in homozygous protein substitutions, we used g:Cocoa [48] to evaluate whether there is an enrichment for proteins that have similar functions. The analysis revealed four significant gene annotations that include a significant number of the queried genes from more than one individual. These four terms are: X-linked recessive inheritance, epithelial cell signaling in *H.pylori* infection, microRNA miR-708 binding sites, and Rho GTPase signaling pathway (Additional file 7: Figure S6).

Although 96 homozygous nsSNPs were identified on the X chromosome, only 1 nsSNP has been documented as a causal variant (X:50658966 *G → A*) (Table 4). Genes involved in X-linked recessive inheritance from the 12 genome data were identified in one African female and four Caucasian males (Additional file 2: Table S9). We identified only two predicted damaging SNPs. One was found in *TBX22* that is associated with X-linked cleft palate. The other is located in *SYTL5*, a trait gene associated with erectile dysfunction and prostate cancer treatment. The remaining X chromosome variants are predicted as neutral. Two male individuals have the same mutation in the *F9* gene, for which reduced function can result in hemophilia B (HEMB). Unlike hemophilia A, symptoms of HEMB are usually milder or can be asymptomatic. Three male individuals also carry an identical SNP in *FRMD7* gene. Malfunctions of this gene can cause nystagmus congenital X- linked type 1 (NYS1), a condition that appears at birth and up to three months old. The indications are spontaneous and involuntary ocular oscillations. Given that most X-chromosome variants are predicted neutral and there is no indication that either of these individuals have these conditions, it is unlikely that the associated disease or trait will develop.

Finally, we used BioGRID [49] to evaluate enrichment for proteins that form physical interaction networks, and found that one of the 12 individual’s genomes has 7 mutations potentially involved in an unusually high number of contacts (Additional file 8: Figure S7). Three of these proteins (FHL2, STK17A, and DSP) are linked together by other interacting partners. The *FHL2* gene encodes a four and a half LIM domain protein that acts as a molecular transmitter between signaling pathways and transcriptional regulation. The wild type amino acid affected by the homozygous mis-sense variant of the *FHL2* gene is evolutionarily conserved, but the *Arg177Gln* substitution is only predicted to be deleterious by one program. This variant is not predicted to affect protein stability and the protein is not associated with a disease or trait. However, we mention it as an example of how this approach may highlight networks of proteins, where subtle modification of multiple partners may result in cumulative disruption that would lead to disease under a multiplicative burden of rare variants model.

## Conclusions

Intensive resource requirements and the associated costs make it infeasible to experimentally verify the effect of every genetic variation. At this stage of human genome study, *in silico* predictions play an important role in identifying putative functional variants. While a sequence-based approach is the current standard practice for assessing SNP effects, there are still some concerns that sequence conservation alone is not a reliable predictor of deleteriousness. In this study, we propose the AACDS classification scheme using variant annotation and sequence-based predictions. We used AACDS to classify homozygous nsSNPs found in the genomes of twelve healthy individuals into eight categories according to the consensus sequence-based deleterious prediction, types of mutation (disease-associated vs. neutral), and information on disease- or trait-associations with the gene. The classification scheme provides a comprehensive framework for prioritizing a list of SNPs suitable for detailed evaluation, in this study reducing the evaluation space from 826 to 98 variants (in categories 1, 2B, 3B, and 4). An online tool for computing the AACDS scores for any variant is provided at http://www.cig.gatech.edu/Tools.

Several previous studies have shown that structural information plays an important role in understanding the relationship between genetic variation and the structure and function of the protein. The addition of 3D structural analysis following AACDS classification demonstrates how structure data can complement sequence-based prediction, and highlights how functional interpretation can in some cases be inferred exclusively from 3D structures. By using a combination of solved structures or high quality homology models for all human proteins, we demonstrate that up to 117 of the 575 proteins bearing homozygous mutations in our CHDWB dataset are available for detailed SNP evaluation, providing detailed analysis of the 150 prioritized variants.

## Competing interests

The authors declare that they have no competing interests.

## Authors’ contributions

TP carried out the analysis. TP and GG participated in the design of the study and wrote the manuscript. Both authors read and approved the final manuscript.

## Supplementary Material

Additional file 1: Figure S1 AACDS summary report. The report is provided to the user with the AACDS category of the variant and its relevant information, along with additional variant data. Click here for file

Additional file 2: Table S1 List of protein structures used in supervised structural analysis. **Table S2.** List of protein structures used in automated structural analysis. **Table S3.** List of all private variants in the 12 genomes. **Table S4.** List of all Categories 2A/2B variants affecting the same gene in more than one individual. **Table S5.** List of all Category 2B variants. **Table S6.** List of all Category 3B variants. **Table S7.** List of all Category 4 Variants. **Table S8.** Summary of automated structural analysis. **Table S9.** List of X-linked recessive mutations. Click here for file

Additional file 3: Figure S2 Cumulative distribution plots for the six deleterious prediction scores. The X-axis represents the prediction scores, ordered by deleteriousness such that low and high scores for each prediction algorithm indicate neutral and damaging nsSNPs, respectively. For each prediction program, the score threshold for defining damaging SNPs is indicated by a vertical green line (threshold for LRT is at 0.999). The genes were classified into four groups depending upon population prevalence of their SNPs, using the difference in minor allele frequencies (MAFs) (cutoff of ± 5%) between European American (EA) and African American (AA) populations. The four gene groups are EA bias, AA bias, EA&AA bias, and no bias. For each plot, the dashed lines illustrate the cumulative distribution of deleterious prediction scores for disease-causing SNPs located in each gene group. The numbers of genes and SNPs are as follows: EA bias (222 genes, 3409 SNPs), AA bias (368 genes, 4825 SNPS), EA&AA bias (234 genes, 4225 SNPs), and no bias (965 genes, 12214 SNPs). All disease-causing nsSNPs were retrieved from MSV3D [14] and SwissVar [15]. Population-specific minor allele frequencies for the variants were derived from NHLBI GO Exome Sequencing Project (ESP6500) (June 2012 release) [12]. Click here for file

Additional file 4: Figure S3 Allele frequency distributions by AACDS score. The three columns indicate the minor allele frequency (MAF) in percent, listed in the order of European American (EA), African American (AA) and all populations (All). Only SNPs with available allele frequency data are represented here and the numbers in each group are 221, 33 and 165, respectively. Click here for file

Additional file 5: Figure S4 Proportions of the 9 types of annotated protein regions found in all residues in the analyzed proteins vs. in SNP residues. Data were compiled from a set of 520 proteins whose sequence features are available from UniProt database [13]. Click here for file

Additional file 6: Figure S5 Location of SNPs within proteins according to sequence feature type. A relative location near zero indicates the SNP is located near the N-terminus of that sequence feature. For clarity, a few features were excluded due to small sample sizes. Click here for file

Additional file 7: Figure S6 Comparison of gene functional enrichment in the 12 genomes. The analysis was performed with g:Cocoa [47]. Each cell in the left most column indicates the number of queried genes from each individual that are associated with each annotation term. The highlighted cells indicate significant enrichment. The enrichment p-values are determined by the default multiple testing correction procedure g:SCS. The column “Term genes” indicate the total number of genes associated to each functional term. Abbreviations: bi: BioGRID protein-protein interaction network; co: CORUM protein complexes; hp: human disease genes from Human Phenotype Ontology; ke/re: KEGG/REACTOME pathway; mi: MicroCosm microRNA sites. Click here for file

Additional file 8: Figure S7 BioGRID network for protein interactions in one person’s genome. The red nodes highlight a subset of the query that is connected by an edge in the network. The black nodes are the immediate neighbors of the red nodes. The one private homozygous nsSNP from this individual is found in the *STK17A* gene. Click here for file
