# Supplementary figures and images for: An association-adjusted consensus deleterious scheme to classify homozygous Mis-sense mutations for personal genome interpretation
Source: BioData Min. 2013 Dec 23;6:24. doi: 10.1186/1756-0381-6-24 (PMC3892026; doi:10.1186/1756-0381-6-24)

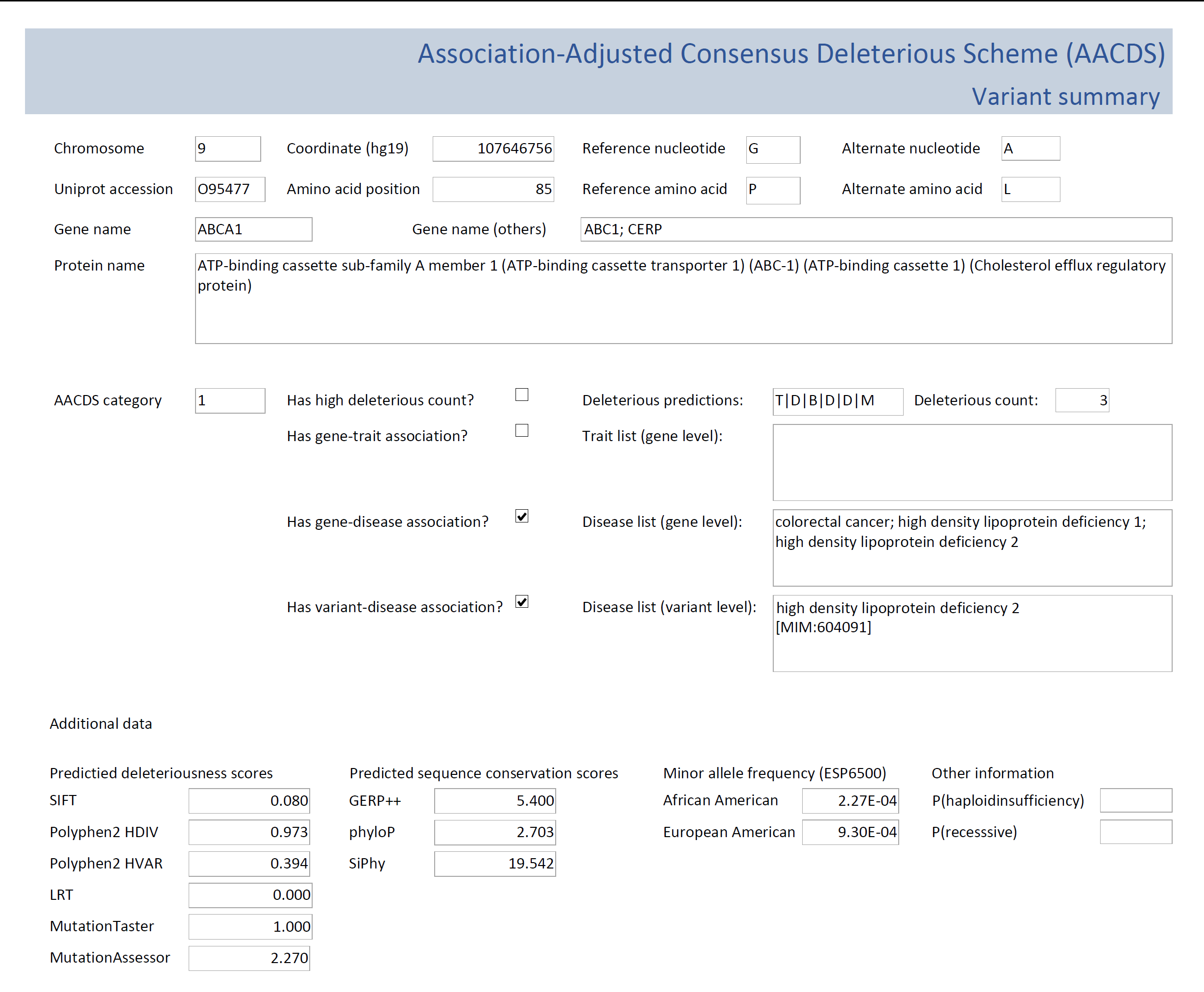

Supplement: Additional file 1: Figure S1 — AACDS summary report. The report is provided to the user with the AACDS category of the variant and its relevant information, along with additional variant data. [file 1756-0381-6-24-S1.png]

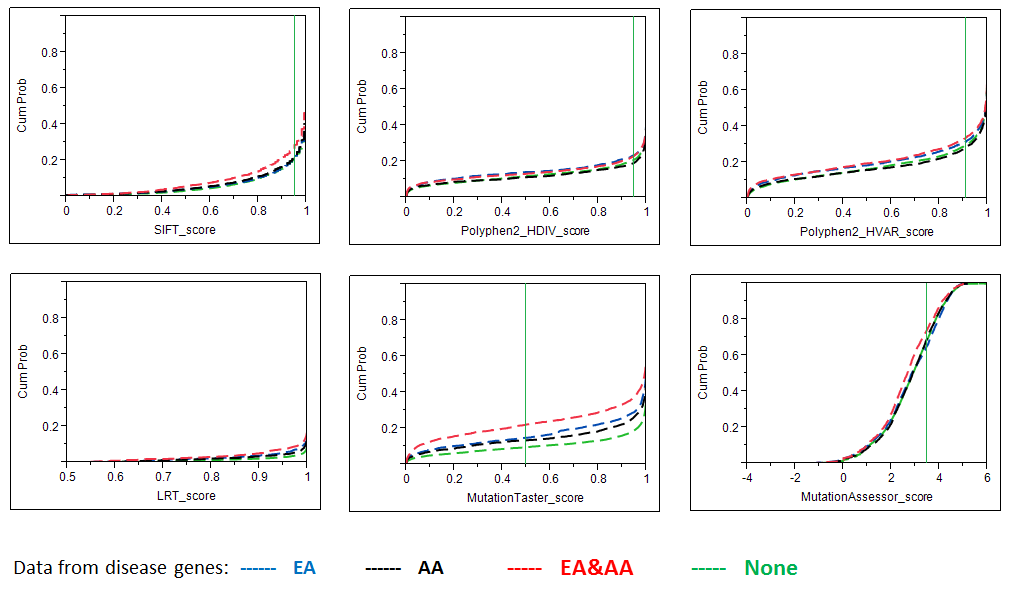

Supplement: Additional file 3: Figure S2 — Cumulative distribution plots for the six deleterious prediction scores. The X-axis represents the prediction scores, ordered by deleteriousness such that low and high scores for each prediction algorithm indicate neutral and damaging nsSNPs, respectively. For each prediction program, the score threshold for defining damaging SNPs is indicated by a vertical green line (threshold for LRT is at 0.999). The genes were classified into four groups depending upon population prevalence of their SNPs, using the difference in minor allele frequencies (MAFs) (cutoff of ± 5%) between European American (EA) and African American (AA) populations. The four gene groups are EA bias, AA bias, EA&AA bias, and no bias. For each plot, the dashed lines illustrate the cumulative distribution of deleterious prediction scores for disease-causing SNPs located in each gene group. The numbers of genes and SNPs are as follows: EA bias (222 genes, 3409 SNPs), AA bias (368 genes, 4825 SNPS), EA&AA bias (234 genes, 4225 SNPs), and no bias (965 genes, 12214 SNPs). All disease-causing nsSNPs were retrieved from MSV3D [14] and SwissVar [15]. Population-specific minor allele frequencies for the variants were derived from NHLBI GO Exome Sequencing Project (ESP6500) (June 2012 release) [12]. [file 1756-0381-6-24-S3.png]

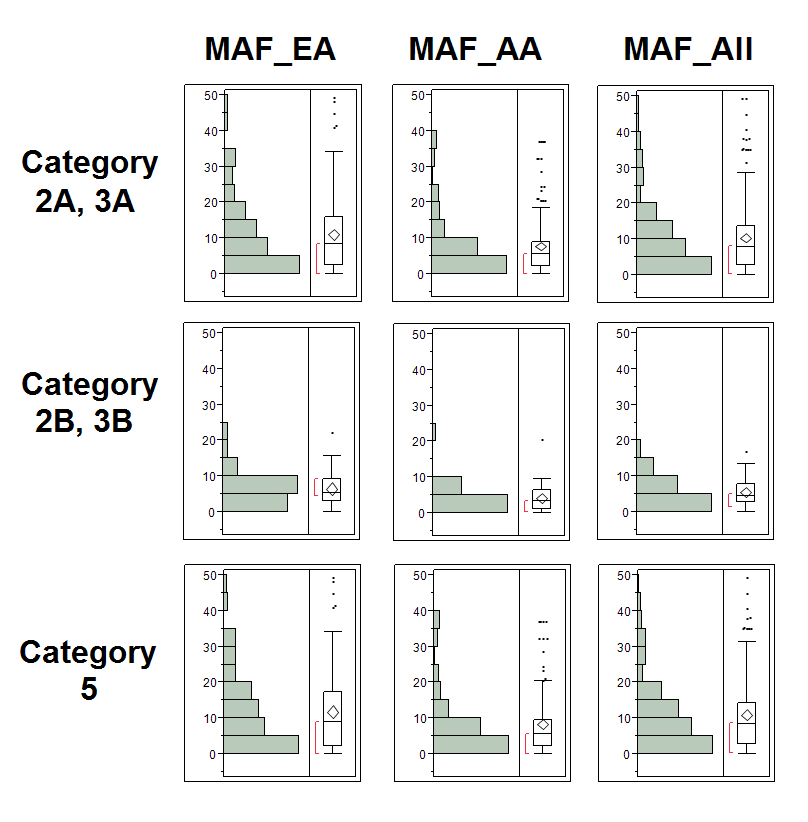

Supplement: Additional file 4: Figure S3 — Allele frequency distributions by AACDS score. The three columns indicate the minor allele frequency (MAF) in percent, listed in the order of European American (EA), African American (AA) and all populations (All). Only SNPs with available allele frequency data are represented here and the numbers in each group are 221, 33 and 165, respectively. [file 1756-0381-6-24-S4.png]

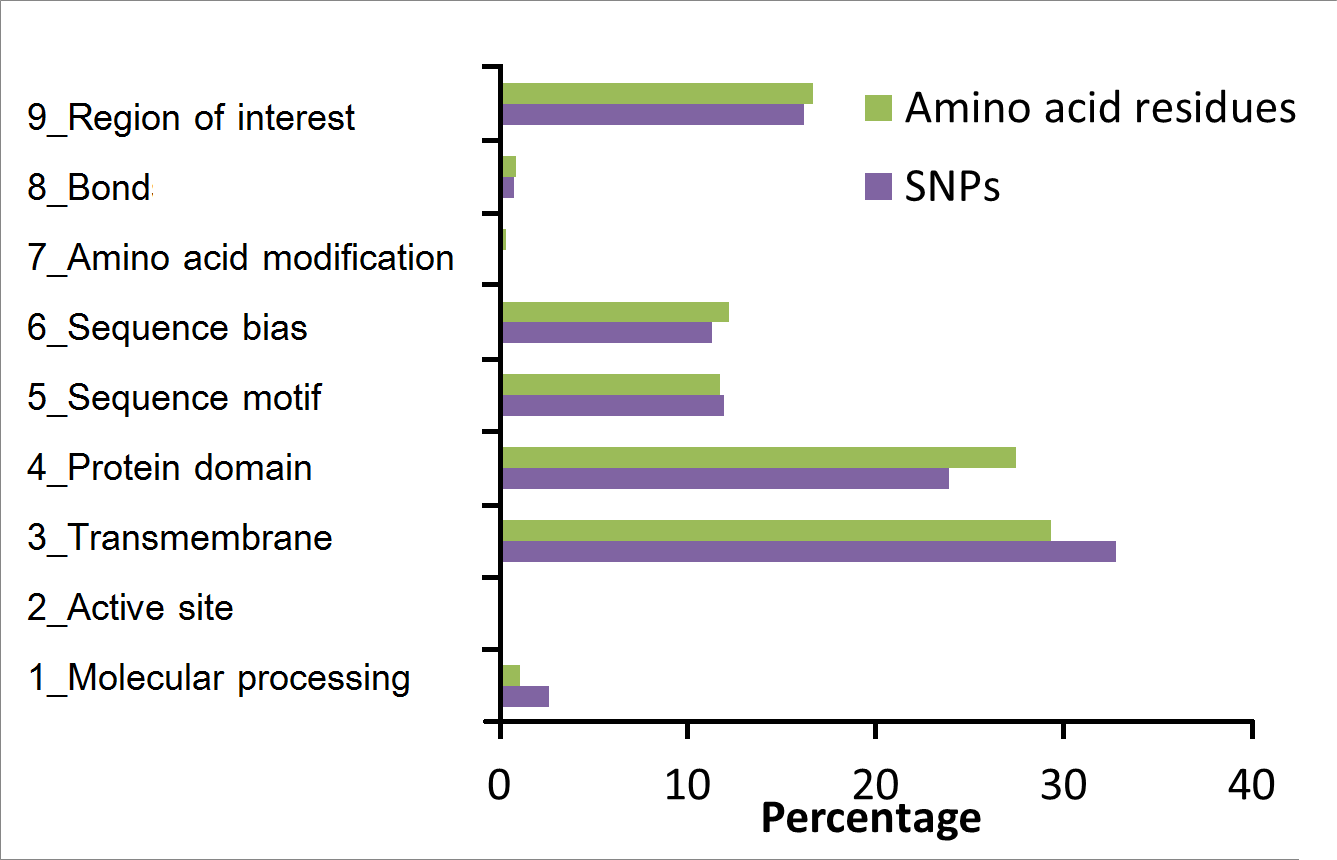

Supplement: Additional file 5: Figure S4 — Proportions of the 9 types of annotated protein regions found in all residues in the analyzed proteins vs. in SNP residues. Data were compiled from a set of 520 proteins whose sequence features are available from UniProt database [13]. [file 1756-0381-6-24-S5.png]

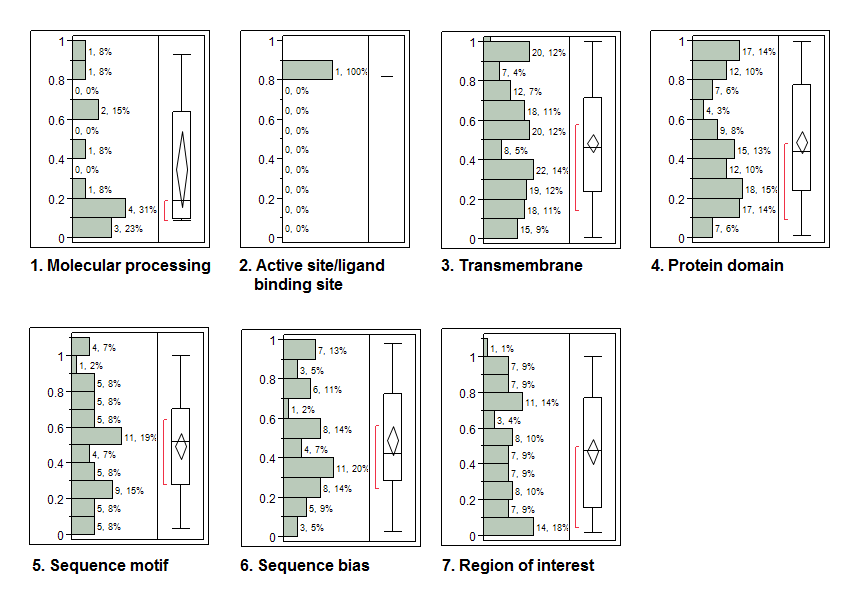

Supplement: Additional file 6: Figure S5 — Location of SNPs within proteins according to sequence feature type. A relative location near zero indicates the SNP is located near the N-terminus of that sequence feature. For clarity, a few features were excluded due to small sample sizes. [file 1756-0381-6-24-S6.png]

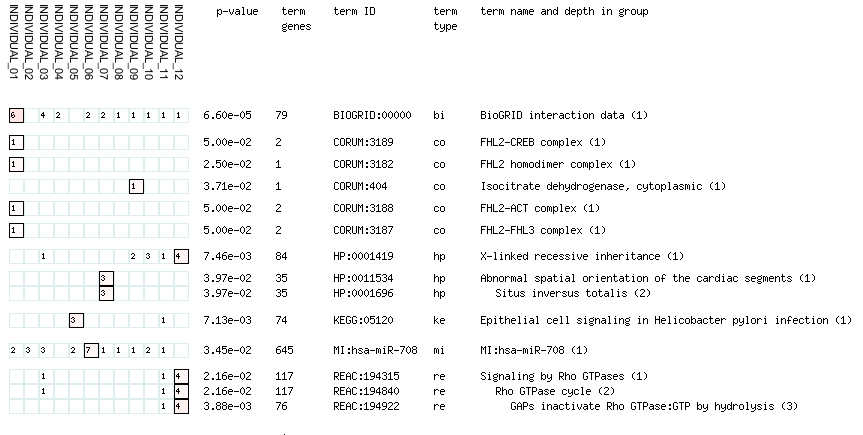

Supplement: Additional file 7: Figure S6 — Comparison of gene functional enrichment in the 12 genomes. The analysis was performed with g:Cocoa [47]. Each cell in the left most column indicates the number of queried genes from each individual that are associated with each annotation term. The highlighted cells indicate significant enrichment. The enrichment p-values are determined by the default multiple testing correction procedure g:SCS. The column “Term genes” indicate the total number of genes associated to each functional term. Abbreviations: bi: BioGRID protein-protein interaction network; co: CORUM protein complexes; hp: human disease genes from Human Phenotype Ontology; ke/re: KEGG/REACTOME pathway; mi: MicroCosm microRNA sites. [file 1756-0381-6-24-S7.png]

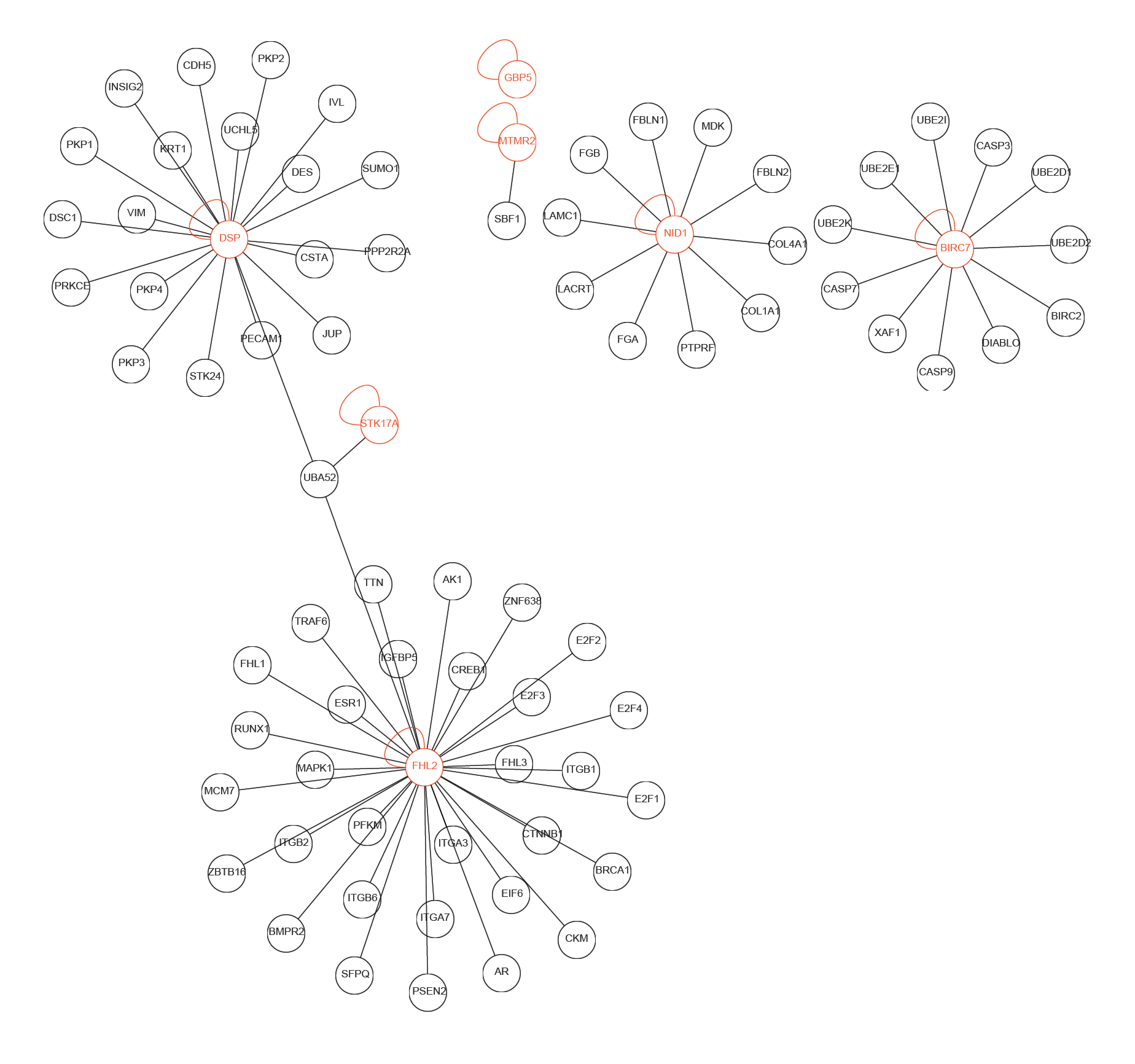

Supplement: Additional file 8: Figure S7 — BioGRID network for protein interactions in one person’s genome. The red nodes highlight a subset of the query that is connected by an edge in the network. The black nodes are the immediate neighbors of the red nodes. The one private homozygous nsSNP from this individual is found in the STK17A gene. [file 1756-0381-6-24-S8.png]
